# Impact of the implementation of a standard for preanalytical handling of samples for microbiological diagnostics on the quality of results at a neurocritical care unit

**DOI:** 10.1097/MD.0000000000027060

**Published:** 2021-08-27

**Authors:** Martin Kieninger, Andreas Mandlinger, Nina Doblinger, Bärbel Kieninger, Sylvia Bele, Bernd Salzberger, Wulf Schneider-Brachert, Bernhard Graf, Florian Zeman, Thomas Holzmann

**Affiliations:** a Department of Anesthesiology, University Medical Center Regensburg, Germany; b Department of Infection Prevention and Infectious Diseases, University Medical Center Regensburg, Germany; c Department of Neurosurgery, University Medical Center Regensburg, Germany; d Center for Clinical Studies, University Medical Center Regensburg, Germany.

**Keywords:** antibiotic stewardship, microbiological diagnostics, neurocritical care, quality of results, standard operating procedure

## Abstract

Antibiotic stewardship (ABS) programs intend to improve outcomes of nosocomial infections and to counteract the emergence of further antimicrobial resistances. At the anesthesiologic-neurosurgical intensive care unit (ICU) of the University Medical Center Regensburg (Germany) we implemented a standard operating procedure (SOP) with clear instructions for the preanalytical handling and storage of microbiological samples. We intended to find out whether the instructions given in the SOP led to a higher rate of ideal material being sent to the laboratory and to overall better quality of the received results.

We retraced retrospectively all samples taken in cases of suspected pneumonia, urinary tract infection, bloodstream infection, catheter infection associated with a central venous or arterial catheter and ventriculitis due to external ventricular drainage as well as all smears taken for the screening for multi-resistant bacteria within a time period of 1 year before to 1 year after the implementation of the SOP.

In the case of suspected pneumonia and urinary tract infection, large amounts of ideal material were sent to the microbiological laboratory. A remarkable improvement after the implementation of the SOP, however, could only be observed regarding the number of urine samples taken from older urinary catheters, which was significantly lower in the “SOP group”. Samples for microbiological diagnostics were taken much more often in the daytime, although storage of the probes did not lead to worse results.

Concrete instructions enable adequate preanalytical handling of microbiological probes. However, we could not recognize substantial improvements probably due to a preexisting high process quality on the ICU. Microbiological diagnostics during the night shift has to be improved.

## Abstract 2

Supplemental Digital Content is available in the text

## 1 Introduction

Manifestation of nosocomial infections is associated with increased mortality and decreased home discharge in neurocritical care patients.^[1]^ The rate of nosocomial infections in this patient population is presumed to be about 30%.^[2,3]^ This emphasizes the importance of adequate microbiological diagnostics and the resulting anti-infective therapies.

Antibiotic stewardship (ABS) programs aim for a targeted use of antibiotics, not only to improve the outcome of infectious diseases in hospitalized patients but also to counteract the emergence of further antimicrobial resistances. Microbiological diagnostics is an essential determinant for achieving the goals of ABS programs,^[4–7]^ although this aspect has often been accorded only limited importance to date.^[8]^ The present study was initiated as part of an ABS program.

The quality of results of microbiological diagnostics is significantly influenced by the preanalytical handling of the samples. This important aspect is often addressed only marginally in the present guidelines or in ABS programs in general, although the demand of precise instructions regarding microbiological laboratory diagnostics seems to be of great importance considering the limited knowledge of technical aspects.^[9]^ A particular potential for improvement should be given for microbiological laboratory diagnostics in cases of suspected urinary tract infections.^[10,11]^

With the objective of improving the preanalytical handling of microbiological samples, a standard operating procedure (SOP) containing clear instructions, for example concerning the selection of proper sample material or the handling of samples in the case the transport to the laboratory is delayed, got implemented at the anesthesiologic-neurosurgical intensive care unit (ICU) of the University Medical Center Regensburg (Germany).

The aim of the present study was to investigate whether the implementation of the SOP had a notable positive effect on the quality of results of microbiological samples.

## 2 Material and methods

The anesthesiologic-neurosurgical intensive care unit of the University Medical Center Regensburg is attended by physicians from the department of anesthesiology and the department of neurosurgery. The primary patient population is made up of postoperative or postinterventional patients of the departments of neurosurgery and neuroradiology, as well as patients with spontaneous intracranial hemorrhage or craniocerebral trauma. But within the framework of the occupancy concept for intensive care patients at the University Medical Center Regensburg also patients from other disciplines are accepted if necessary.

**Figure 1:**
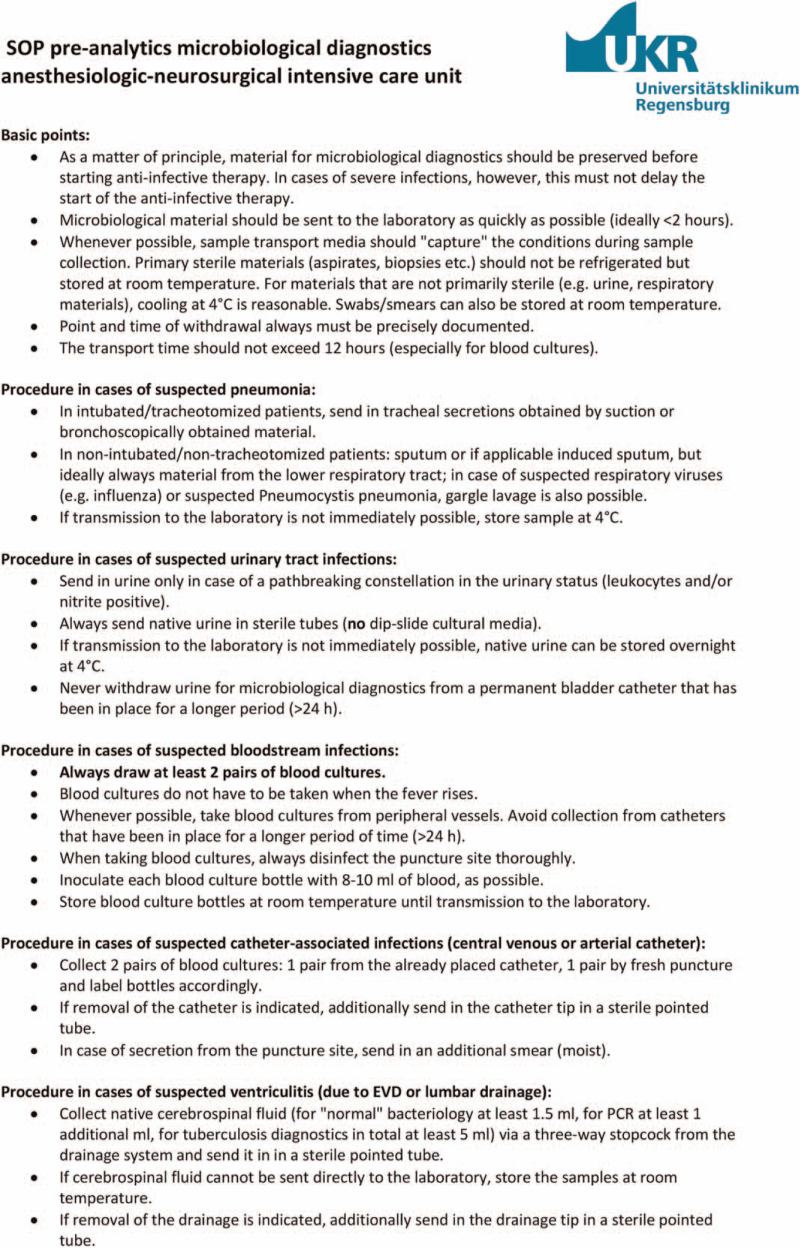
Standard operating procedure (SOP) for the preanalytical handling of microbiological samples. EVD = external ventricular drainage, PCR = polymerase chain reaction.

On May 1, 2018 an SOP for the preanalytical handling of microbiological samples for the diagnostics for bacterial and mycotic infections was implemented (Fig. 1). This SOP was created in collaboration with the infectious disease specialists and microbiologists of the hospital, who supervise the intensive care unit in regular medical rounds. Prior to the implementation of the SOP, no written standard was available regarding the preanalytical handling of microbiological probes. Because of their participation in an ABS program at the hospital, however, the ICU staffs have been receiving feedback from the microbiological laboratory in the case of obviously incorrectly handled samples for many years.

At the University Medical Center Regensburg, microbiological samples can only be immediately sent to the in-house laboratory of the department of microbiology during specific time periods. These range from 8:00 a.m. to 17:30 p.m. on weekdays and from 8:00 a.m. to 13:30 p.m. on weekends and public holidays. Samples collected outside these working hours will only be able to be processed immediately in justified exceptional cases, when a medical emergency is on hand, but will normally have to be temporarily stored at the intensive care unit until the following day.

### 2.1 Study design

Following a retrospective study design with a pre-post comparison all microbiological samples sent from the anesthesiologic-neurosurgical intensive care unit to the institute of microbiology and hygiene in cases of suspected pneumonia, urinary tract infection, bloodstream infection, infection associated with a central venous or arterial catheter and ventriculitis due to external ventricular drainage (EVD) as well as all smears taken for the screening for multi-resistant bacteria were retraced within a time period of 1 year before to 1 year after the implementation of the SOP. This way, a “historical group” including the samples taken prior to the implementation of the SOP (May 1, 2017–April 30, 2018) and a “SOP group” with samples taken afterwards (May 1, 2018–April 30, 2019) were generated. An evaluation of routinely stored data and documents in 3 software applications took place: The patient data management system of the intensive care unit (patient data management system, iMDsoft, Tel Aviv, Israel), the clinical SAP system (Walldorf, Deutschland) and the system for statistics and analytics of the hospital hygiene (HyBASE, epiNET AG, Bochum, Deutschland).

### 2.2 Exclusion criteria

Samples taken from children and adolescents under the age of 18 years were excluded from the analysis. Similarly, postoperative and postinterventional patients with uncomplicated clinical course and a length of stay at the intensive care unit of less than 24 hours were not considered in the analysis.

### 2.3 Ethics

The study was conducted in accordance with the approval from the local ethics committee (18-1028-101).

### 2.4 Statistical analysis

Data were statistically analyzed using software (IBM SPSS Statistics Version 26, IBM, Armonk). Metric data were presented as mean ± standard deviation provided that normal distribution was given, or as median and interquartile range (IQR) provided that not. According to the given distribution of data, testing among the groups was performed using Student *t* test or Mann–Whitney *U* test. Categorical variables were indicated as frequency and comparison between the groups was performed using Chi-Squared-test. *P* values below .05 were considered statistically significant. In this retrospective study, no a priori sample size calculation was performed. Instead, all patients available in the pre-defined period were included to maximize the power of the study.

## 3 Results

### 3.1 General data

**Figure 2:**
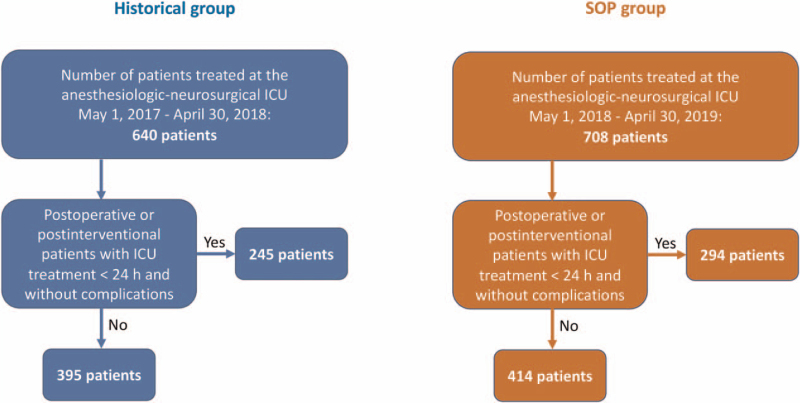
Flow chart for patient enrollment. SOP = standard operating procedure, ICU = intensive care unit.

During the observation period 640 patients in the historical group and 708 patients in the SOP group were treated at the anesthesiologic-neurosurgical intensive care unit of the University Medical Center Regensburg. In the historical group 245 patients (38.3%) and in the SOP group 294 patients (41.5%) were excluded from further analysis due to the character of their stay being solely postoperative or postinterventional, of less than 24 hours duration and without any complications. Therefore 395 cases in the historical group and 414 cases in the SOP group remained (Fig. 2). Demographic data of these patients and data regarding ICU treatment are presented in Table 1. The patients in the historical group were slightly younger than the patients in the SOP group with a median of 61 years (IQR 49–70 years) and 63 years (IQR 50–76 years), respectively (*P* = .012). The patients of both groups had the same median length of stay of 4 days (IQR 1–11 days in the historical group, IQR 2–10 days in the SOP group, *P* = .871). Neurosurgical patients still made up the biggest part of both groups after subtraction of the above mentioned uncomplicated postoperative cases (historical group 273 patients, 69.1% and SOP group 265 patients, 64.0%, *P* = .124).

**Table 1:** Demographic data of the patients.

	Historical group (n = 395)	SOP group (n = 414)	*P* value
Median age [IQR] (yrs)	61 [49–70]	63 [50–76]	.012
Sex (m/f)	243/152	244/170	.453
Median length of ICU stay [IQR] (days)	4 [1–11]	4 [2–10]	.871
Percentage of NS patients	69.1%	64.0%	.124
Percentage of GS patients	3.1%	3.9%	.393
Percentage of VS patients	1.3%	1.4%	.822
Percentage of ENT/OMF patients	4.2%	5.1%	.605
Percentage of CTS patients	1.7%	1.2%	.507
Percentage of IDM patients	10.8%	10.6%	.906
Percentage of IM patients	8.6%	11.6%	.159
APercentage of TS patients	1.2%	2.2%	.322

CTS = cardiothoracic surgery, ENT/OMF = ear, nose and throat medicine/oral and maxillofacial surgery, f = female, GS = general surgery, ICU = intensive care unit, IDM = interdisciplinary medicine (particular affiliation not possible due to more than one leading diagnosis in one patient), IM = internal medicine, IQR = interquartile range, m, male, NS = neurosurgery, SOP = standard operating procedure, TS = trauma surgery, VS = vascular surgery. Significant *P* values are displayed in bold.

### 3.2 Microbiological diagnostics in cases of suspected pneumonia

A total of 139 samples from the patients’ respiratory tract were sent to the microbiological laboratory in the historical group and 93 such samples were sent in the SOP group. In every case the material had been obtained correctly in accordance with the SOP. The frequency of positive findings did not differ between the 2 groups observed: 75 out of the 139 samples in the historical group (54.0%) vs 56 out of the 93 samples in the SOP group (60.2%, *P* = .246). In 44 cases of the, in total, 75 positive samples of the historical group and in 33 of the 56 positive samples of the SOP group, exactly 1 type of germ was detected (58.7% vs 58.9%, *P* = .976). Two types were found in 27 samples in the historical and 18 samples in the SOP group (36.0% vs 32.1%, *P* = .646). And 3 or more types were merely detected in 4 and 5 samples (5.3% vs 9.0%, *P* = .421). Thirty three of the 139 samples of the historical group (23.7%) and 28 of the 93 samples of the SOP group (30.1%) had to be temporarily stored prior to transportation to the microbiological laboratory. Even when only looking at these cases separately, the frequency of positive findings did not differ between the 2 groups (17 positive samples, 51.5%, in the historical group vs 16 positive samples, 57.1%, in the SOP group, *P* = .660). An overview over the detected germs and their frequency taken all respiratory tract samples from the 2 groups together is provided in Supplement S1, http://links.lww.com/MD2/A350.

### 3.3 Microbiological diagnostics in cases of suspected urinary tract infection

In case of a suspected urinary tract infection, in total 24 samples in the historical group and 36 samples in the SOP group were sent to the microbiological laboratory. In all of these cases, correctly, native urine was gathered rather than using dip-slide cultural media. But, in the SOP group significantly more frequently either fresh urine or urine from a urinary catheter having been inserted within the past 24 hours reached the laboratory (5 out of 24 samples i.e., 20.8% in the historical group vs 21 out of 36 samples i.e., 58.3% in the SOP group, *P* = .001). In the historical group 12 and in the SOP group 16 urine samples were sent to the microbiological laboratory although either no urinalysis had existed beforehand or a urinalysis had been conducted but showed no typical signs of a urinary tract infection (50.0% vs 44.4%, *P* = .673). The frequency of positive findings regarding microbiological analysis of the urine did not differ between the 2 groups (16 out of 24 samples i.e., 66.7% in the historical group vs. 19 out of 36 samples i.e., 52.8% in the SOP group, *P* = .285). Solely 1 pathogenic germ was found in 10 samples of the historical and 15 samples of the SOP group (62.5% vs 78.0%, *P* = .283). Two types of pathogenic germs could be detected in 4 samples of both the historical and the SOP group (25.0% vs 21.1%, *P* = .782). Three or more types of pathogenic germs were cultivated from the material of 2 samples of the historical group, but of no sample of the SOP group (12.5% vs 0%, *P* = .112). Also, when looking only at those cases, where the urine could not be sent immediately to the laboratory but had to be temporarily stored (4 samples of the historical and 5 samples of the SOP group), the frequency of positive findings did not differ in a statistically significant manner (3 samples, 75.0%, in the historical group vs 2 samples, 40.0%, in the SOP group, *P* = .294). The frequency of the detected germs in the urine samples of both groups is shown in Supplement S2, http://links.lww.com/MD2/A351.

### 3.4 Microbiological diagnostics in cases of suspected bloodstream infection

Blood cultures for diagnostics in cases of suspected bloodstream infection were taken 33 times in the historical group (total number of blood cultures sent to the microbiological laboratory 65) and 58 times in the SOP group (total number of blood cultures sent to the microbiological laboratory 115). Transmittal of correct material (at least 2 pairs of bottles and no blood withdrawal out of vascular catheters older than 24 hours) was rare in both groups (8 out of 33 cases i.e., 24.2% in the historical group vs 16 out of 58 cases i.e., 27.6% in the SOP group, *P* = .782). In contrast, blood cultures were frequently taken from vascular catheters having been introduced longer than 24 hours ago (34 out of 65 blood cultures i.e., 49.2% in the historical group, 55 out of 115 blood cultures i.e., 47.8% in the SOP group, *P* = .564). In all transmitted blood cultures, germs could be identified in 13 out of the 65 samples in the historical group and in 17 out of the 115 samples in the SOP group (20.0% vs 14.8%, *P* = .367). Among these, 1 type of pathogenic germ was found in 11 of the 13 positive blood cultures of the historical group and in 15 of the 17 blood cultures of the SOP group (84.6% vs 88.2%, *P* = .773). Two types of pathogenic germs had been cultured from 2 samples of the 2 groups (15.4% vs 11.8%, *P* = .773). None of the analyzed blood cultures showed 3 or more types of germs. Temporary storage was necessary in 22 of the 65 blood cultures (33.8%) of the historical group and in 28 of the 115 blood cultures (24.3%) of the SOP group. These cases showed no difference in the frequency of positive findings among the 2 groups (5 samples i.e., 22.7% in the historical group, 7 samples i.e., 25.0% in the SOP group, *P* = .852). The identified germs from all positively tested blood cultures are shown in Supplement S3, http://links.lww.com/MD2/A352 together with their frequency.

### 3.5 Microbiological diagnostics in cases of suspected vascular infection associated with a central venous or arterial catheter

In the historical group, 25 cases of suspected vascular infection associated with a central venous or arterial catheter led to the transmittal of material to the microbiological laboratory (in total 58 individual samples), in the SOP group 11 cases (in total 23 individual samples). Correct material (at least 1 pair of blood cultures taken from the respective catheter together with at least 1 pair inoculated with freshly drawn blood, plus additional transmittal of the catheter tip when the catheter was removed) was obtained in 10 of the 25 cases in the historical and in 5 of the 11 cases in the SOP group (40.0% vs 45.5%, *P* = .760). Twenty two of the 58 individual samples of the historical group and 7 of the 23 samples of the SOP group were found to be positive (37.9% vs 30.4%, *P* = .526). Of these, in 18 cases in the historical group and in 5 cases of the SOP group only 1 type of germ was detected (81.8% vs 71.4%, *P* = .554). Two types of germs were cultured from 4 and 1 sample, respectively (18.2% vs 14.3%, *P* = .812), while only 1 sample from the SOP group contained 3 different types of germs.

16 of the 58 samples of the historical group (27.6%) and 9 of the 23 samples of the SOP group (39.1%) had to be temporarily stored prior to their transmission to the laboratory. Here, germs were detected in 8 samples of the historical and 3 samples of the SOP group (50.0% vs 33.3%, *P* = .420). In Supplement S4, http://links.lww.com/MD2/A353 the cultured germs and their frequency in the cases of suspected catheter-associated infection are depicted.

### 3.6 Microbiological diagnostics in cases of suspected ventriculitis associated with external ventricular drainage

Material from patients with the suspected diagnosis of ventriculitis due to EVD was sent to the microbiological laboratory in 28 cases in the historical group (30 individual samples) and in 27 cases in the SOP group (27 individual samples). Correct material (native cerebrospinal fluid in a sterile conical tube, plus additional transmittal of the drainage tip when removing the drainage) reached the laboratory in 18 cases of both groups (64.3% vs 66.7%, *P* = .853). Positive findings resulted from 4 samples of the historical and 5 samples of the SOP group (13.3% vs 18.5%, *P* = .592). In both groups, each positive sample always showed only 1 type of germ. 4 samples of the historical as well as the SOP group had to be temporarily stored at the intensive care unit. In the historical group, a pathogenic germ was cultivated from 2 of these samples, in the SOP group from 1 (50.0% vs 25.0%, *P* = .465). The positive samples of both groups contained Staphylococcus hominis in 4 cases, Staphylococcus epidermidis in 3 cases and methicillin-sensitive Staphylococcus aureus in 2 cases.

### 3.7 Smears to screen for multi-resistant bacteria

Smears to screen for multi-resistant bacteria requiring patient isolation were taken from various patients prior to transferring them to an external hospital or rehabilitation center. In the historical group, 490 samples were taken from 119 patients in this context, in the SOP group 603 samples from 156 patients. Comparing the frequency of positive findings between the 2 groups, the level of significance was just missed (11 positive samples in the historical group i.e., 2.2%, 5 positive samples in the SOP group i.e., 0.8%, *P* = .053). Supplement S5, http://links.lww.com/MD2/A355 portrays the detected multi-resistant germs from the samples of both groups.

## 4 Discussion

Ventilator-associated pneumonia has the highest prevalence within nosocomial infections in critically ill neurosurgical patients according to a recently published large prospective cohort study.^[12]^ The same result was obtained by a study on hospital-acquired infections in patients after intracerebral hemorrhage published in 2016.^[1]^ In this context it seems reasonable that respiratory material was transmitted most frequently for microbiological diagnostics. The correct collection of samples of respiratory material from intubated ICU patients is easy to realize and not associated with any relevant expenditure of time such as in the case of blood cultures taken from newly inserted central venous catheters. In this context, the large amount of correctly sampled respiratory material sent to the laboratory led to an overall high quality in microbiological testing. The finding that more than 50% of the samples in the historical group were positive does not seem to be surprising because it was probably not possible to further improve the quality of preanalytical handling of respiratory probes.

Urinary tract infections are the second most frequent entity of nosocomial infections in neurosurgical patients.^[1,12]^ But regarding microbiological diagnostics in cases of potential catheter-associated urinary tract infections, caution should be taken to prevent unindicated diagnostics and needless antibiotic therapy.^[13,14]^ Accordingly, the SOP included the direction to only send urine to the microbiological laboratory if the clinical conditions of the patient as well as the urinalysis raised suspicion. In contrast, analysis of both groups revealed that in about 50% of the cases microbiological diagnostics of urine got initiated without reasonable suspicion. The high rate of non-adherence to the specifications of the SOP is difficult to understand because unnecessary microbiological testing is associated with unnecessary expenditure of time and costs. A possible explanation could be that serious infections of unclear etiology—which often occur in critically ill patients at the ICU—are still often followed by wide-ranging but unspecific microbiological diagnostics. The sense of this approach, however, has to be questioned critically. So, in this regard further training seems to be indicated. An important improvement in the SOP group was that urine was less frequently taken from urinary catheters that had been inserted more than 24 hours before. However, this did not have a measurable impact on the quality of results. Thus, more than half of the samples of both groups resulted in positive findings and mostly only 1 type of pathogenic germ was detected at a time.

Looking at microbiological diagnostics in cases of suspected bloodstream infection, most of the time non-ideal material reached the microbiological laboratory both before and after implementation of the SOP. Likewise, in the case of suspected catheter-associated vascular infection, ideal material was sent to the laboratory in only about 50% of patients of both groups. Blood cultures from fresh venipuncture were lacking in most cases. A possible explanation for the obvious disregard of the guidelines could be the considerable additional time and effort occasionally needed for fresh venipuncture compared to the withdrawal from directly inserted catheters. However, it is generally surprising that besides the fact that blood cultures were often taken from vascular catheters inserted some time before, the rate of false-positive findings was apparently low in both groups. Nevertheless, correct blood sampling is essential to provide the best conditions for high-quality microbiological diagnostics, despite the possible additional expenditure of time and the fact that no clear effect on the quality of the results could be proven in the present study.

Development of meningitis or ventriculitis is a potential life-threatening major complication after the insertion of an EVD. The literature presents varying data regarding the incidence. In a large retrospective cohort study involving 34,238 patients a rate around 7% was determined.^[15]^ Transmission of ideal material for microbiological diagnostics in this context should be feasible with little time and complications, which was confirmed by our data. The rate of positive findings was very low in both groups. Limitingly, it needs to be mentioned that prior to planned implantations of ventriculo-peritoneal shunts, cerebrospinal fluid or the tip of an EVD was rather often sent to the microbiological laboratory for diagnostics, even in the absence of reasonable suspicion of infection. This was due to requests from the surgeon in charge. In all of the positively cultivated samples only one type of germ could be found at a time reflecting a high quality of microbiological diagnostics. At our anesthesiologic-neurosurgical ICU, we are highly experienced in the treatment of critically ill patients with an inserted EVD. Handling of the EVD, early recognition of infectious complications, and specifications for treatment in the case of suspected infection of the cerebrospinal fluid are part of the initial training of all physicians and nurses. In this context, it seems understandable that the process quality of microbiological diagnostics for suspected infection of the cerebrospinal fluid was already high before the implementation of the SOP, so that no further improvement could be achieved. Otherwise, it would not be comprehensible to exclude cerebral infections in an SOP designed for a neurocritical care unit.

It is generally noteworthy that microbiological samples were mainly obtained during the operating times of the microbiological laboratory and only rarely during the night. Taken the assumption that the manifestation of an infection will occur independently of the time of day and should entail immediate response in form of microbiological sample collection and start of an anti-infective therapy as a basis, 1 must conclude that the quality of infection treatment in the intensive care unit is inferior at night. A possible cause for the rare nightly collection of samples for microbiological diagnostics may have been the assumption that a temporary storage of the samples could result in inferior quality of results. However, the results of the presented study do not confirm this fear, independently of the analyzed material. Furthermore, the clear instructions in the SOP regarding storage of the samples if immediate transmission is not possible should eliminate any uncertainty. Given the fact that infections in critically ill patients with severe courses always have to be suspected when a further worsening of patients’ condition occurs, an adequate treatment including microbiological sample collection for diagnostics prior to the start of an anti-infective therapy must be ensured regardless of the time of day and without delay. Here we see an urgent need for action. However, it is not possible to determine from the available data whether only the microbiological diagnostics was carried out with delay or not at all, or whether there was even a delay in the demanded initiation of anti-infective therapy. This question could be the subject of a further study.

### 4.1 Limitations

The main limitation of the present study is that the evaluation of the data was carried out retrospectively. Information on the quality of the results of the microbiological samples is limited because of the study design. Poor result quality may be assumed in situations highly suspicious for bacterial infection (e.g., infiltrations in chest X-ray diagnostics together with compromised pulmonary gas exchange, high white blood cell counts and positive nitrite in urine, or high cell counts and low glucose levels in cerebrospinal fluid) when cultivation of a pathogen fails or when numerous pathogens are cultivated, especially in urine or cerebrospinal fluid samples or in blood cultures. The degree of adherence to the instructions of the SOP on the modalities of storing samples that could not be directly sent for microbiological diagnostics remains unclear. The undetectable effect on the quality of results in these samples could therefore be due to the same treatment of the samples of the 2 groups. A further important point could have contributed to the lack of significant differences between the 2 groups: because of their participation in an ABS program, the ICU staff had regularly received feedback from the microbiological laboratory in the case of incorrectly handled samples for several years. This feedback is likely to have continuously improved process quality, despite the lack of an SOP on this topic at the time.

## 5 Conclusion

The aspect of preanalytical sample handling in microbiological diagnostics should be given higher priority in ABS programs. The creation of an SOP can be helpful in this regard. In the present study, however, hardly any relevant effects could be shown by the implementation of such an SOP. A noteworthy aspect was that microbiological samples were mainly obtained in the daytime during the acceptance periods of the bacteriological laboratory. We see an urgent need for action here, since it must be assumed that nosocomial infections in intensive care units manifest themselves independently of the time of day. As a matter of principle in the case of suspected infection, the aim should be to immediately preserve specific material for microbiological diagnostics and then start a calculated anti-infective therapy right away.

## Author contributions

**Conceptualization:** Martin Kieninger, Bernd Salzberger, Thomas Holzmann.

**Data curation:** Bärbel Kieninger, Thomas Holzmann.

**Formal analysis:** Martin Kieninger, Florian Zeman.

**Investigation:** Andreas Mandlinger.

**Methodology:** Florian Zeman, Thomas Holzmann.

**Project administration:** Martin Kieninger, Thomas Holzmann.

**Software:** Florian Zeman.

**Writing – original draft:** Martin Kieninger, Nina Doblinger.

**Writing – review & editing:** Nina Doblinger, Bärbel Kieninger, Sylvia Bele, Bernd Salzberger, Wulf Schneider-Brachert, Bernhard Graf, Florian Zeman, Thomas Holzmann.

## Supplementary Material

SUPPLEMENTARY MATERIAL
