# Supplementary material for: Impact of the implementation of a standard for preanalytical handling of samples for microbiological diagnostics on the quality of results at a neurocritical care unit
Source: Medicine (Baltimore). 2021 Aug 27;100(34):e27060. doi: 10.1097/MD.0000000000027060 (PMC10545238; doi:10.1097/MD.0000000000027060)
Supplement: SUPPLEMENTARY MATERIAL [file medi-100-e27060-s001.pdf]

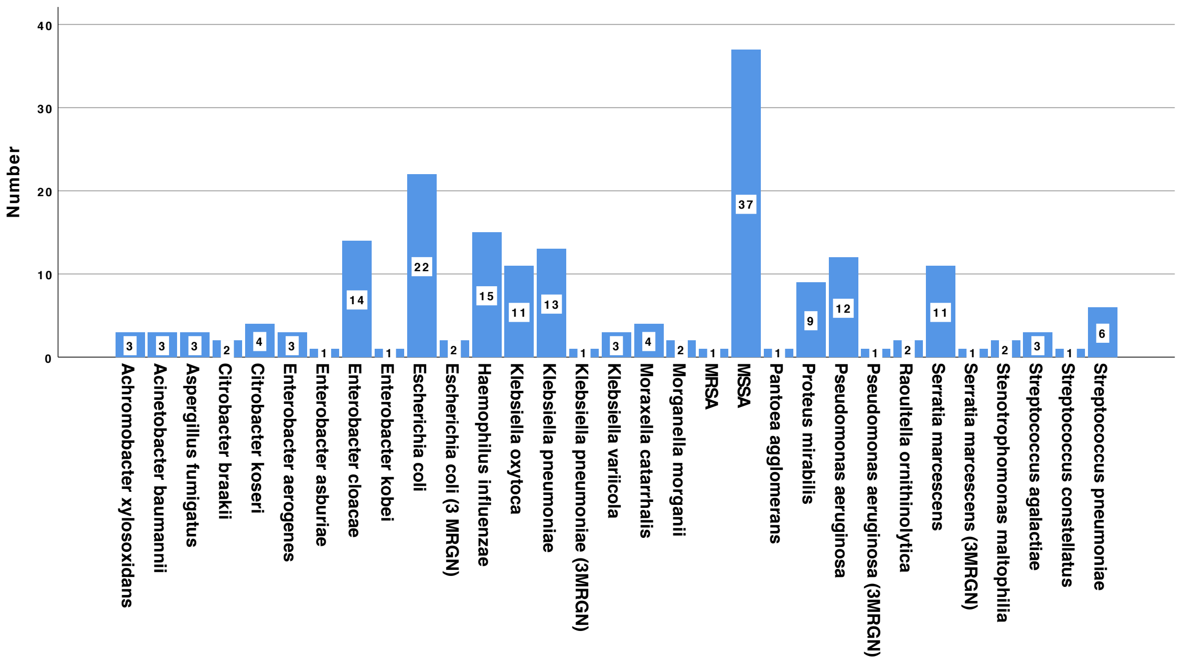

Detected germs and their frequency in all respiratory material taken from the patients of the historical as well as the SOP group. MRSA = methicillin-resistant Staphylococcus aureus, MSSA = methicillin-sensitive Staphylococcus aureus, MRGN = multidrug-resistant gram-negative bacteria.
